# Six-Minute Walk Test: Reference Values and Prediction Equation in Healthy Boys Aged 5 to12 Years

**DOI:** 10.1371/journal.pone.0084120

**Published:** 2013-12-31

**Authors:** Nathalie Goemans, Katrijn Klingels, Marleen van den Hauwe, Stefanie Boons, Liese Verstraete, Charlotte Peeters, Hilde Feys, Gunnar Buyse

**Affiliations:** 1 Department of Child Neurology, University Hospitals Leuven, Leuven, Belgium; 2 Department of Rehabilitation Sciences, KU Leuven, Leuven, Belgium; Charité Universitätsmedizin Berlin, NeuroCure Clinical Research Center, Germany

## Abstract

**OBJECTIVE:**

This study aimed to (1) generate normative data in healthy boys aged 5–12 years for the six-minute walk test (6MWT), an outcome measure currently used in clinical trials in Duchenne muscular dystrophy (DMD), (2) to describe the relation with anthropometric variables and myometry, and (3) to compare our data with published equations.

**METHODS:**

The 6MWT was conducted in 442 boys according to a standardized protocol, as currently used in clinical trials in DMD. Maximal voluntary isometric contractions for knee flexion and extension were recorded with a hand-held myometer.

**RESULTS:**

The 6MWD increased significantly with age, from 478.0±44.1 m at age 5, to 650.0±76.8 m at age 12, with the steepest increase between 5 and 8 years. Age- and height related percentile curves of the 6MWD were developed. Correlations with anthropometric variables were fair to good (age r = 0.60, height r = 0.57, weight r = 0.44). Myometric variables (knee flexors and extensors) showed correlations of 0.46 and 0.50 respectively. When dividing into two age categories (5–8 years, 9–12 years), these magnitudes of correlations only applied to the younger age group. Additionally, predicted values were calculated according to available reference equations (Geiger and Ben Saad), indicating an overestimation by those equations. Finally, the Geiger equation was refitted to our population.

**CONCLUSION:**

The percentile curves according to age and height provide a useful tool in the assessment of ambulatory capacity in boys aged 5 to 12 years. Significant correlations with anthropometric variables and myometry were only found in the 5–8 years age group. The Geiger prediction equation, currently used to assess ambulatory capacity in DMD was refitted to obtain a more accurate prediction model based on a large sample with a homogenous distribution across the age categories 5 to 12 years and applying the methodology as currently used in clinical trials in DMD.

## Introduction

The six-minute walk test (6MWT), a measure of function and endurance originating from the cardiorespiratory field, assesses the distance a subject is able to walk in six minutes at a normal pace (six-minute walking distance, 6MWD) [1]. This measure reflects the physical capacity and walking function at a submaximal level and has been accepted as a clinically meaningful outcome measure by the regulatory authorities in registration-directed clinical trials in neuromuscular and neurometabolic disorders [2].The 6MWT, slightly modified from the original American Thoracic Society guidelines, has been validated in Duchenne Muscular Dystrophy (DMD), a severe X linked progressive disorder, and is currently used as (primary) endpoint in therapeutic trials in ambulatory boys with DMD [3]. Moreover, the correlation of 6MWT with the Pediatric Outcomes Data Collection Instrument (PODCI), a Quality of Life (QoL) instrument score, has been reported recently in DMD [4], confirming its applicability to detect clinically meaningful changes in ambulant DMD.

The evolution of the 6MWD in DMD is characterized by a specific pattern: despite of the degenerative character of this disease, recent studies showed an increment in the 6MWD up to approximately the age of 7 followed by a decline which becomes precipitous around the age of 12 years [5]–[8]. In order to account for maturational influences, it has been suggested to convert raw data of the 6MWD into percent predicted values based on normative data to describe the natural evolution or the impact of interventions in this disease against the background of growth and development [9]. This method is commonly applied for other outcome measures such as respiratory function [10], [11].

Different studies have reported normative data and prediction equations for the 6MWT in healthy children from different ethnic, environmental and geographical backgrounds [12]–[20]. However, data on 6MWT are scarce in young boys of Western European descent in the age range and according to the procedures currently used in clinical trials in DMD. Moreover, study methods and testing procedures differ from one study to another, with different track lengths and testing instructions or pooling of data from both genders, hampering further comparison [12]–[27]. Furthermore, recent studies indicated that published equations may over- or underestimate 6MWD when applied to other populations [12], [15], [18], [19], highlighting that further research is warranted to validate the existing equations in large samples of typically developing children, applying standardized procedures currently used in DMD trials.

Predictive factors for the 6MWD have been explored such as age and anthropometric variables, underscoring the influence of age and height on this outcome measure [12]–[20]. However, the impact of other variables such as muscle strength has not yet been reported in children.

To address those issues, we investigated the 6MWD in typically developing young boys. In a previous study, we reported on the reliability of this outcome measure and the developmental evolution of the 6MWT in narrow age subcategories in boys between age 5 and 12 years, an age range of particular interest for clinical trials in ambulant DMD [28]. In this current study, the data set was further expanded to a larger cohort in order to generate reference values and percentile curves for the 6MWT in typically developing boys of this age. A second aim was to investigate the relation of this functional capacity measure with age, anthropometric variables and underlying muscle strength. Finally, we evaluated the applicability of published reference equations to our population.

## Methods

### Participants

Typically developing boys aged 5–12 years were recruited from five randomly selected local primary schools in Belgium between January and May 2012. Children had to be able to understand and fully comply with the assessments. A questionnaire to identify health related problems was completed by the parents a few days before testing. Children with known chronic cardiac, respiratory, neurological or musculoskeletal disorders were excluded. Participants were sampled across eight age subcategories with one year interval between 5.0 and 13.0 year and six height subcategories with 10 cm interval from 105 cm on. The final sample included also 90 boys from our previously reported reliability study recruited based on the same in- and exclusion criteria and who completed the 6MWT according to the same procedure [28].

### Ethics statement

Ethical approval was obtained from the institutional committee of the University Hospitals Leuven (Commissie voor medische ethiek/klinisch onderzoek UZ Leuven) and the institutional boards of the participating schools (Basisschool Sint-Jozef Coloma Mechelen, Vrije Basisschool Putte,Vrije Basisschool De Bunderboog Moorslede, Gemeentelijke Basisschool Moorslede, Sint-Martinus Basisschool Lubbeek, Heilig Hart Instituut Heverlee, Don Bosco Heverlee, Sint-Romboutscollege Mechelen). Parents of all children gave their written informed consent.

### Test Procedure

The participants' weight and height were determined using standardized anthropometric methods before the testing. The 6MWT was conducted according to a standardized protocol, as described by McDonald et al. [3]. The test was performed in a flat, straight corridor. Each boy walked for six minutes counterclockwise at his preferred pace along a 25 m tape line, with cones placed at each end of the course. Evaluators gave a standardized demonstration prior to the test. Subsequently, one practice trial over one track length was done to ensure that the child understood the instructions. During the test, each boy was followed by a ‘safety chaser’ giving limited standardized encouragements.

Testing was performed by three physiotherapists experienced with the test procedure.

### Myometry

Knee flexion and extension strength was measured in both legs using a calibrated MicroFET2 handheld myometer and expressed in Newton (N).Testing was performed in a standardized starting position, sitting with hips and knees in 90° of flexion and no feet contact with the floor. The investigator fixated the femur above the knee and placed the handheld myometer at the posterior part of the calcaneus for knee flexion and at the anterior part of the shin just proximally to the ankle joint for knee extension [29]. The highest value of three maximum isometric contractions was recorded in knee flexors and extensors bilaterally by using the ’ ‘make’ technique which requires the patient to exert a maximal isometric contraction while the examiner holds the dynamometer in a fixed position.

To rule out the influence of left-right differences, summed scores for left and right leg were calculated.

### Statistical analysis

Descriptive statistics were applied for the different variables within the total sample and within each age and height group. Data were tested for normality by Shapiro-Wilk tests and graphically checked for symmetry.

To construct percentile curves, the following percentiles of 6MWT were estimated for different values of age and height using a quantile regression analysis: 5%, 10%, 25%, 50%, 75%, 90% and 95%. The interior algorithm was used to estimate the regression parameters. The explanatory variables age and height were included using restricted cubic splines so that no assumptions had to be made regarding the type of association with the results of the 6MWT. Correlation analysis between 6MWD and age, anthropometric, and myometric variables was performed in the subgroup of 352 subjects in whom myometric data were available. Pearson product-moment correlation coefficients (r) were calculated for the total group and for two age categories, from 5 to 8 and 9 to 12 years. Correlation coefficients of >0.70 were considered as high, between 0.50–0.70 good, between 0.30–0.50 fair and of <0.30 weak or no association [30].

Measured 6MWDs were compared with the distances predicted based on the published reference equations by Geiger et al. in Austria [14] (6MWD (m) = 196.72+39.81× age (years) −1.36×age^2^ (years)+138.28× height (m)) and Ben Saad et al. in North-Africa [12] (6MWD (m) = 4.63× height (cm) −3.53× weight (kg)+10.42× age (years)+56.32) using parametric paired t-tests and scatterplots.

Finally, a refitted Geiger model was obtained by applying the Geiger model to 100 bootstrap samples and averaging the regression coefficients across all bootstrap samples to obtain the final estimate of the regression coefficients.

All analyses were performed using SAS software, version 9.2 and SAS Enterprise Guide.

## Results

### Participant characteristics

In the current study, 368 medical questionnaires were filled out correctly and returned by the parents. Sixteen children were excluded based on one or more exclusion criteria (chronic cardiovascular, respiratory or motor disorders). A total of 442 boys, including the 90 subjects of the first study [28], were included in the analysis. All boys were of Western European (98%) or Northern African (2%) descent. Anthropometric data, age, 6MWD and velocity per age and height subcategory are reported in Table 1. Mean age, height and weight of the total group were 9.0±2.3 years, 135±14.16 cm and 31.5±9.63 kg respectively.

**Table 1:** Participants characteristics, six-minute walk distance and velocity according to age and height categories (Mean values ± standard deviation).

		N	Age (y)	Height (cm)	Weight (kg)	Distance (m)	Velocity (m/min)
Age	5 years	57	5.6±0.3	115±5.69	20.7±2.5	478.0±44.1	79.7±7.4
	6 years	52	6.5±0.3	121±6.13	23.6±3.2	516.1±61.8	86.0±10.3
	7 years	56	7.5±0.3	127±4.41	25.8±3.4	559.2±65.4	93.2±10.9
	8 years	55	8.5±0.3	133±5.93	29.0±4.2	604.3±72.0	100.7±12.0
	9 years	60	9.5±0.3	139±5.61	32.8±6.0	595.7±69.0	99.3±11.5
	10 years	53	10.5±0.3	144±7.28	35.5±6.2	633.1±70.0	105.5±11.6
	11 years	61	11.4±0.3	148±6.73	39.5±7.8	625.9±83.0	104.3±13.8
	12 years	48	12.5±0.3	154±6.74	46.7±7.3	650.0±76.8	108.3±12.8
Height	105–114 cm	43	5.7±0.4	111±2.43	19.4±1.9	468.6±46.5	78.1±7.8
	115–124 cm	70	6.6±0.9	120±2.83	23.1±1.9	529.0±65.6	88.2±10.9
	125–134 cm	104	8.0±1.1	129±3.09	27.3±3.6	576.5±67.8	96.1±11.3
	135–144 cm	100	9.8±1.2	140±2.83	32.7±4.2	605.3±73.3	100.9±12.2
	145–1541 cm	89	11.2±0.7	149±2.64	40.6±6.5	631.6±80.2	105.3±13.4
	>155 cm	36	12.1±0.7	159±4.65	49.3±7.1	651.2±88.3	108.5±14.7
TOTAL		442	9.0±2.3	135±14.16	31.5±9.63	582.2±88.2	97.0±14.7

### 6MWD and velocity

Descriptive data of 6MWD and velocity are given in Table 1.The overall mean 6MWD was 582.2±88.2 m. Mean 6MWD increased between the age of 5 and 12 from 478.0±44.1 m to 650.0±76.8 m. In parallel, velocity increased from 79.7±7.4 m/min at 5 years to 108.3±12.8 m/min at 12 years. The steepest increase was observed between the age of 5 and 8 years (from 478.0 m to 604.3 m). Beyond this age, the 6MWD tended to stabilize (from 604.3 m to 650.0 m). In the height subcategories, distance increased significantly across the board with an increase of mean 6MWD from 468.6±46.5 m in the smallest boys up to 651.2±88.3 m in the tallest boys with velocities of respectively 78.1±7.8 m/min to 108.5±14.7 m/min. A mild flattening was seen from a height of 140 cm.

### Percentile curves according to age and height

**Figure 1:**
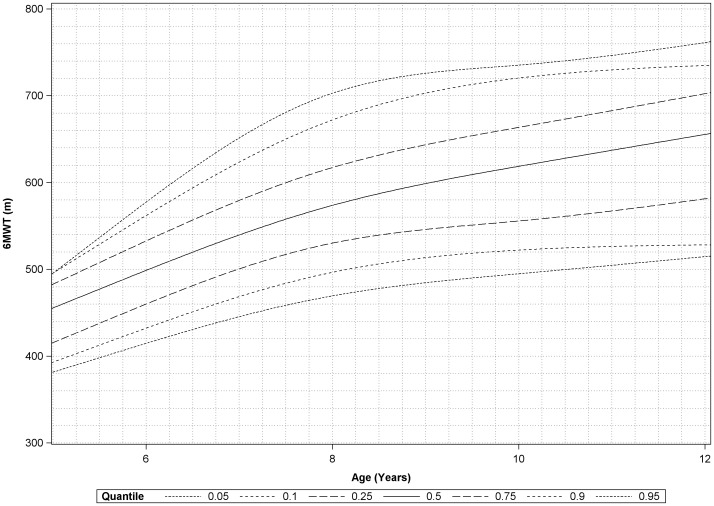
Plot of estimated percentiles of six-minute walk test versus age. Percentiles 5%, 10%, 25%, 50%, 75%, 90% and 95% of 6MWT were estimated for different values of age.

**Figure 2:**
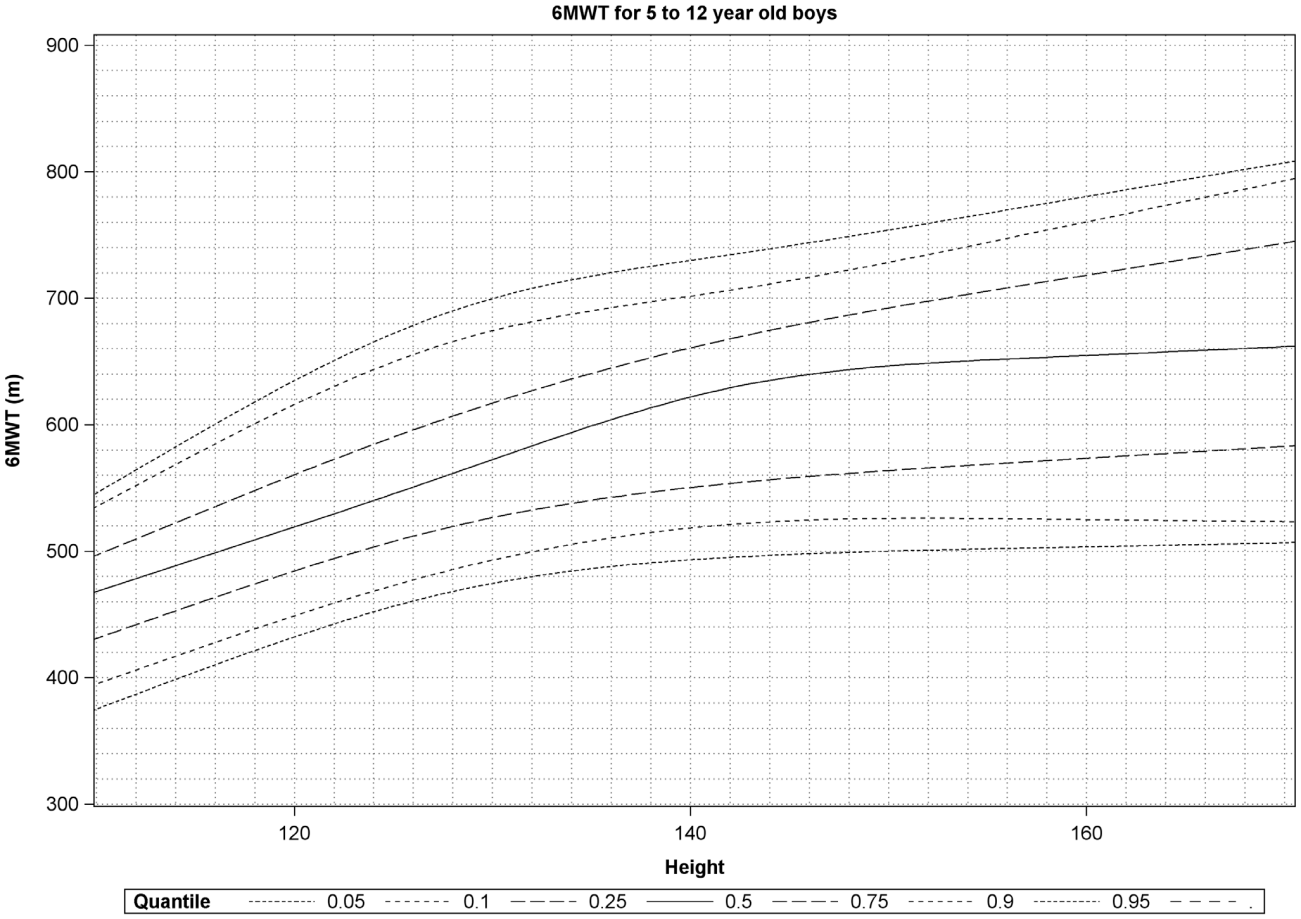
Plot of estimated percentiles of six-minute walk test versus height. Percentiles 5%, 10%, 25%, 50%, 75%, 90% and 95% of 6MWT were estimated for different values of height.

Percentiles 5%, 10%, 25%, 50%, 75%, 90% and 95% of 6MWT were estimated for different values of age and height and are shown in Figures 1 and 2.

### Myometry

Myometry data were obtained in 352 subjects. The mean muscle strength ± SD for the total group for the knee flexors and extensors was 189.2±53.9N and 346.8±112.7N, respectively (Table 2). Muscle strength increased with age for both knee flexors and extensors. Myometric variables were also calculated for the two age cohorts of 5 to 8 and 9 to 12 years.

**Table 2:** Mean values (standard deviation) of myometric variables for the total group and according to eight and two age categories.

	Total group	Eight age categories								Two age categories	
	**5–12 years**	**5 years**	**6 years**	**7 years**	**8 years**	**9 years**	**10 years**	**11 years**	**12 years**	**5–8 years**	**9–12 years**
**N**	352	42	42	43	45	46	44	48	42	172	180
					**Myometric variables**						
**Flexors**	189.2	127.0	143.0	168.4	182.0	194.1	219.4	214.7	260.2	155.6	221.2
**(N)**	(53.9)	(27.1)	(28.3)	(30.5)	(25.0)	(32.5)	(45.5)	(45.9)	(48.8)	(34.9)	(49.2)
**Extensors**	346.8	221.1	250.3	301.6	328.5	366.8	417.4	405.5	472.3	276.5	414.1
**(N)**	(112.7)	(39.3)	(55.0)	(55.0)	(65.4)	(97.2)	(87.1)	(97.3)	(121.4)	(65.8)	(107.0)

### Correlations of 6MWD with age, anthropometric and myometric variables

Correlations between 6MWD and predictive variables were calculated for the total group and for the two age subcategories (5 to 8 and 9 to 12 years) (Table 3). For the total group, height and weight were significantly correlated with the 6MWD (r = 0.57; r = 0.44 respectively, p<0.0001). Age showed the highest correlation with the 6MWD (r = 0.60; p<0.0001).

**Table 3:** Correlation coefficients between six-minute walk distance and anthropometric and myometric variables for the total group and for two age categories with statistical comparison.

	Total group	Two age categories		z-value	p-value
	5–12 years	5–8 years	9–12 years		
**N**	352	172	180		
**Age**	0.60*	0.65*	0.16*	5.71	p<0.0001
**Weight (kg)**	0.44*	0.50*	−0.02	5.29	p<0.0001
**Height (cm)**	0.57*	0.60*	0.09	5.61	p<0.0001
**Flexors (N)**	0.46*	0.46*	0.08	3.88	p = 0.0001
**Extensors (N)**	0.50*	0.52*	0.16*	3.86	p = 0.0001

p<0.05

The 6MWD showed a fair to good correlation with knee flexion (r = 0.46; p<0.05), and knee extension (r = 0.50; p<0.05). Inspection of the correlation coefficients in the two age subcategories revealed a clear difference between both groups. In the younger children, significantly higher correlations were found between all variables and 6MWD, compared to the older children. Anthropometric factors showed a good correlation with the 6MWD in the young age category, with coefficients above 0.50 while in the older age group, no to weak associations were found. The same trend was observed for the myometric variables, showing fair to good correlations in the younger age category (r = 0.46 – 0.52) and weak or no association with 6MWD in the older age group.

### 6MWD reference equation

Table 4 compares our results to mean values of previous 6MWT studies in healthy children from different countries and ethnicities. Differences in methodology between these studies are summarized in table 5.Overall, data measured in Austrian [14] and North African boys [12] were higher in comparison with the present study. In contrast, measured data in children from U.S, U.K and South America were clearly lower [15], [16], [20].

**Table 4:** Measured 6MWD of the present study and comparison with reported and predicted 6MWD.

	5 years	6 years	7 years	8 years	9 years	10 years	11 years	12 years
**Measured 6MWD of Present study (m) Belgium**	478.0±44.1	516.1±61.8	559.2±65.4	604.3±72.0	595.7±69.0	633.1±70.0	625.9±83.0	650.0±76.8
**Predicted 6MWD by Geiger et al. (14) (m)**	527.6±11.1	556.9±12.1	586.9±7.7	611.9±9.1	635.4±8.5	655.6±11.2	670.2±9.6	685.5±9.7
**Predicted 6MWD by Ben Saad et al. (12) (m)**	566.3±20.6	595.9±21.1	624.3±17.2	652.7±18.3	677.0±18.2	703.4±25.4	718.8±23.0	730.5±29.6
**Measured 6MWD of Geiger et al (14) (m) Austria**	536. 5±95.6 (3–5y)		577.8±56.1 (6–8 y)			672.8±61.6 (9–11 y)		697.8±74.7 (12–14y)
**Measured 6MWD of Ben Saad et al. (12) (m) North Africa**		543±33 (6–7 y)		667±55 (8–9y)		715±31 (10–11 y)		725±68 (12–13y)
**Measured 6MWD of Klepper et al (15) (m) United States**			534.5±60.3 (7–8 y)		515.8±81.4	497.9±74.0	534.9±88.9	
**Measured 6MWD of Lammers et al (16)** * **(m) United Kingdom**	420±39	463±40	488±35	483±40	496±53	506±45	512±41	
**Measured 6MWD of Priesnitz et al (20)** * **(m) South America**		508.3±54.0	550.2±61.6	556.7±67.2	594.2±60.6	602.4±61.1	608.0±54.3	618.1±51.4

*All measured 6MWD are reported for males, except Lammers et al. and Priesnitz et al. who reported mean distances for males and females together.

**Table 5:** Comparison between methodologies of reported 6MWT studies in healthy children from different countries and ethnicities.

Study	Country	Sample size Total (M/F)	Age range		Age categories			Distance	Modifications to ATS	Gender
**Geiger et al (14)**	Austria	528 (280/248)		3–18		3–5, 6–8, 9–11, 12–15, >16		20 m	Measuring wheel, 3–4 year old allowed to run/jog	Separate for male and female
**Ben Saad et al (12)**	North Africa	200 (100/100)	6–16		6–7, 8–9, 10–11, 12–13, 14–15, 16			30 m	Best of two tests (60 min interval), no encouragement	Separate for male and female
**Klepper et al (15)**	United States	100 (43/57)	7–11		7–8,9,10,11			15–25 m	Two tests (15 min interval), standardized encouragements	Separate for male and female
**Lammers et al (16)**	United Kingdom	328 (177/151)		4–11		4,5,6,7,8,9,10,11	15–25 m		Continuous HR and SaO2 measurement by follower	Male + female
**Priesnitz et al (20)**	South America	188 (92/96)	6–12		6,7,8,9,10,11,12			30 m	First of two tests (30 min interval), standardized encouragements	Male + female
**Present study**	Belgium	442 (Male)	5–12		5,6,7,8,9,10,11,12			25 m	According to McDonald et al (3) (safety chaser, instructions, standardized encouragements)	Only male

A comparison with previously published reference equations was performed by calculating predicted values for our measured data based on the reference equations of Geiger et al. [14] and Ben Saad et al. [12]. Mean differences between our actual measured values and the predicted values by Geiger et al.[14] and Ben Saad et al. [12] were −33.7 m (95% CI 27.3 m – 40.1 m) and −76.1 m (95% CI 69.5 m – 82.6 m) respectively, indicating a significant difference (both p<0.0001).

### Comparison with Geiger prediction model

**Figure 3:**
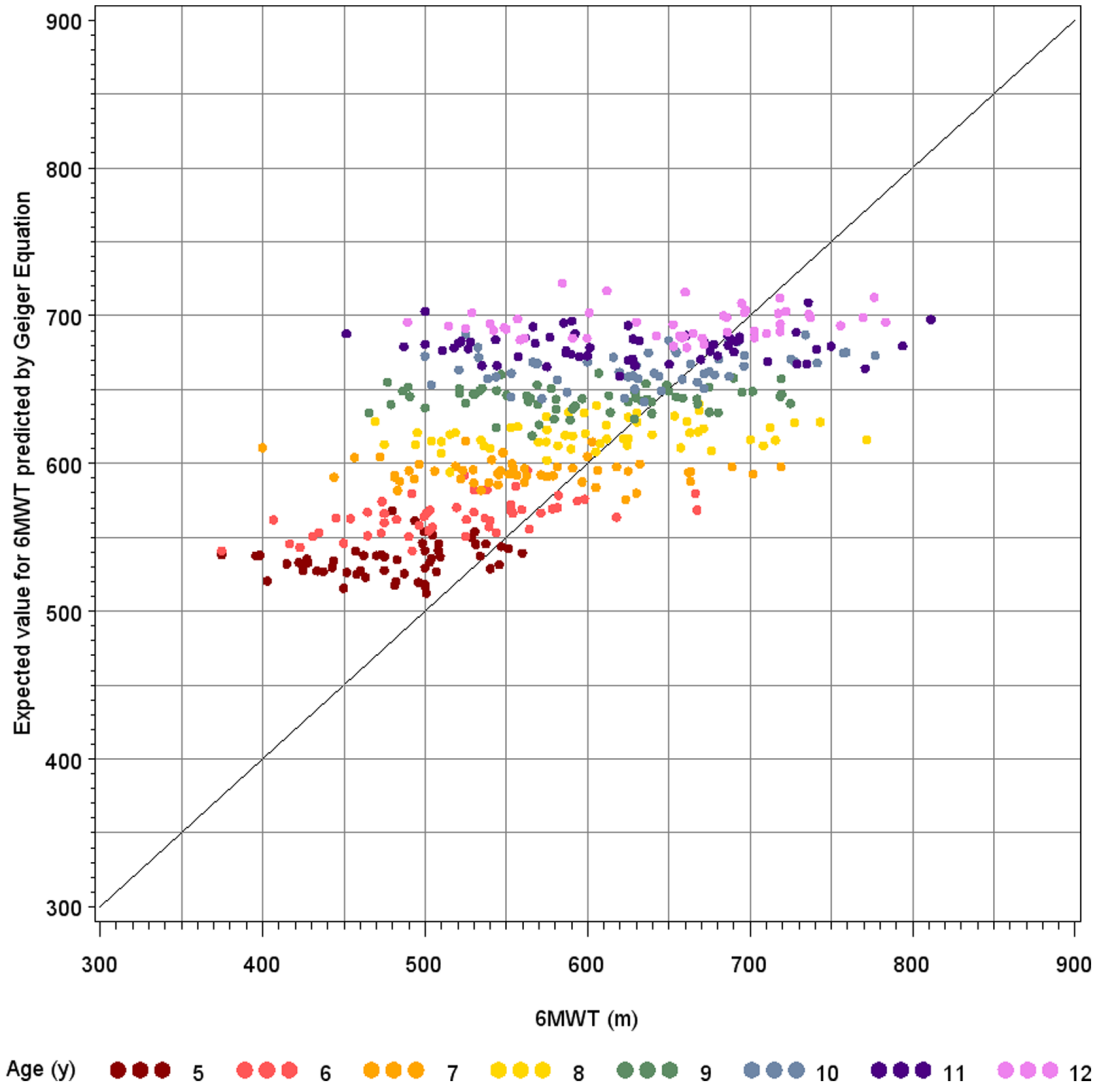
Predicted versus observed six-minute walk test data for the Geiger equation. The observed 6MWT data were plotted against the predicted data based on the Geiger equation applied to our study sample (R^2^ = 0.17).

Figure 3 illustrates the observed 6MWD versus the predicted values based on the Geiger equation. This application resulted in a systematic overestimation with an associated R^2^ value of 0.17. Therefore the Geiger model was refitted for our population with resulting R^2^ value of 0.41. The following refitted Geiger equation was obtained:

6MWD = 86.795 + 74.547× age (years) −3.018× age^2^ (years) +63.204× height (m).

**Figure 4:**
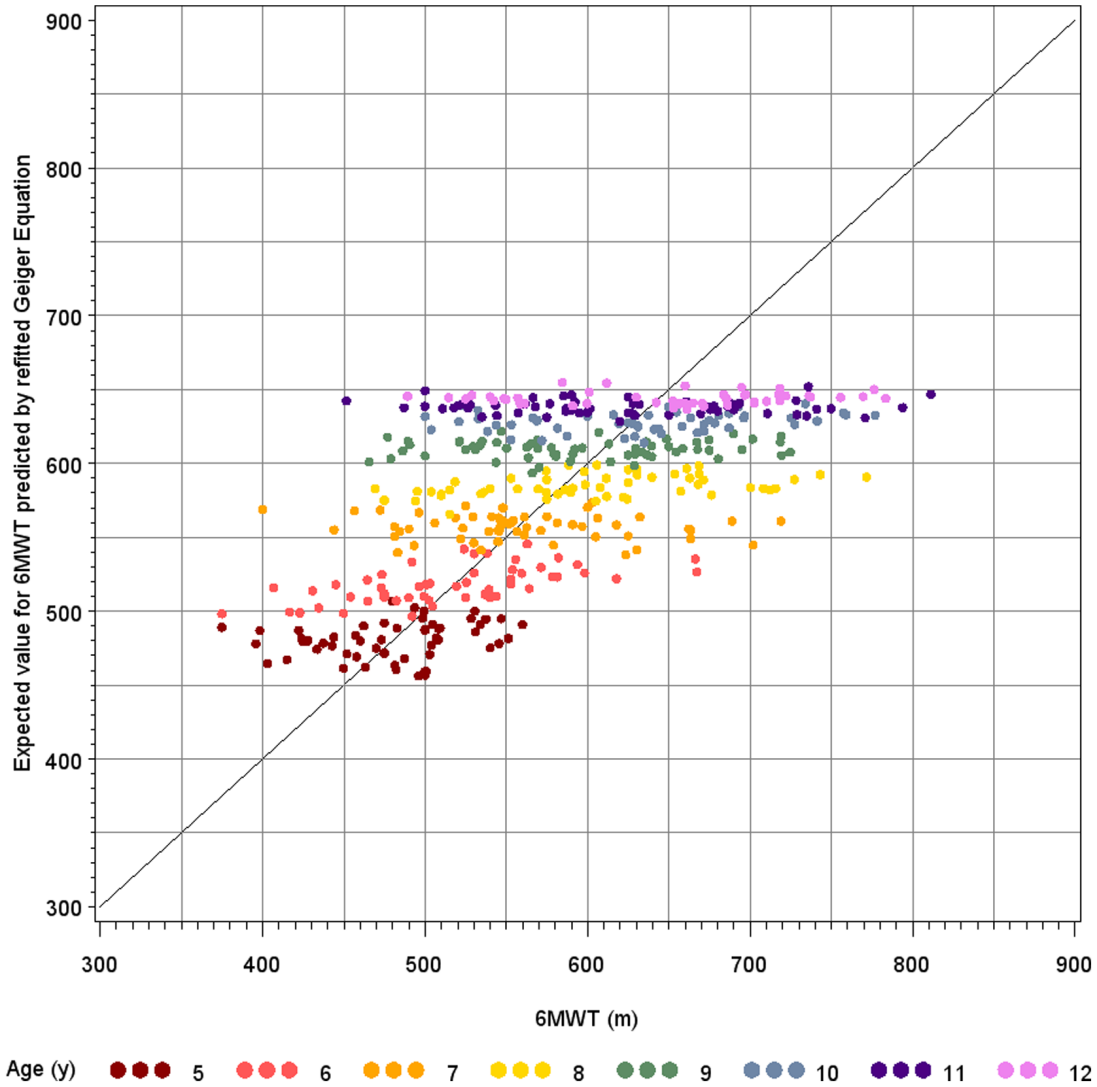
Predicted versus observed six-minute walk test results for the refitted Geiger equation. The observed 6MWT data were plotted against the predicted data based on the refitted Geiger equation applied to our study sample (R^2^ = 0.41).

Figure 4 shows the predicted versus observed 6MWT results for the refitted Geiger equation, indicating a better distribution around the identity line.

## Discussion

This study established normative data for the 6MWT, provided age- and height specific centile curves and investigated its correlation with age, anthropometric and myometric variables in a large cohort of 442 healthy boys aged 5–12 years, an age group of particular interest in relation to studies in ambulant DMD boys. In addition, a comparison with previously published reference data and prediction equations was made and the commonly used Geiger equation was refitted for this population. To our knowledge, this is the largest dataset reporting on 6MWT in typically developing boys of this age range, using the methodology and track length currently applied in DMD studies [3].

Our findings confirmed previous reports of a significant improvement in 6MWD with increasing age [16], [28]. The strongest increase was found between the age of 5 and 8 years, which is in accordance to previous reports from Austrian [14] and British children [16]. After the age of 8 years, 6MWD tends to stabilize despite a further evolution of the anthropometric variables [12], [14], [16], which may possibly be explained by the developmental maturity of the human gait pattern and muscle activation patterns at this age. Moreover, the velocity of the walking pattern has a known physiological limit from approximately 5 km/h [31], [32]. At higher velocity there is a disproportionate increase in energy expenditure, indicating that in older children where a full maturation of gait pattern is achieved, a switch to a running pattern is more economical from an energy standpoint and required to further increase velocity. It should, however, be noted that DMD subjects are typically unable to run due to their muscle weakness and generally show a decline in 6MWD after the age of 8.

A steady increase was noticed over the height subcategories, followed by a flattening of the curve from height 135 cm on, which is in accordance with the data of Lammers et al [16]. Both age- and height specific centiles curves were constructed, which could provide a user-friendly method in the prediction of 6MWD in boys of this age range. We were particularly interested in investigating the relation of the 6MWD with height and to provide height specific references in addition to the age specific normative data, as those could be useful in DMD boys, known to have a stunted growth compared to their peers, which is aggravated by the chronic use of corticosteroids.

Height specific reference centiles have been published based on data from 805 boys and 610 girls from Chinese ethnicity (aged 7–16 years) [17]. Comparison of the reported mean 6MWD and inspection of the centile curves for boys indicated slightly higher values for the Chinese population compared to our sample. This difference could be explained by differences in methodology, with the use of a longer track length in the Chinese study and/or by ethnic differences as suggested by other authors [12], [15], [18].

A second aim was to investigate the correlation of age, anthropometric variables and leg strength with the 6MWD. The highest correlation was found for age and height with correlation coefficients of 0.60 and 0.57 respectively, which confirms previously reported findings [12], [14], [16], [17], [20]. A fair to good correlation was found between 6MWD and muscle strength in knee flexors and extensors. However, these magnitudes of correlations appeared mainly driven by the age group 5 to 8 years and disappeared in the older age group. Discordance between muscle strength and function tests has been reported by other authors [33]. Finally, our data indicated that additional factors to age, anthropometric and myometric variables influence the 6MWD, which requires further investigations, especially in older children. Genetic predisposition, motor abilities, physical fitness and training as well as motivation of the individual child during a self-paced test may further impact on the measured 6MWD.

In addition, we aimed to compare our normative data with previous reports. Literature review revealed eight studies [12]–[18], [20] that reported measured values and/or prediction equations for the 6MWT in healthy children from different ethnic, environmental and geographical backgrounds. Our 6MWD data showed consistently lower values than the measured values of Geiger et al. [14] and Ben Saad et al. [12]. Adversely, measured 6MWDs in North American [15], British [16], and South American boys [20] were lower than our measured values. However, a direct comparison of our findings to the reference values reported should be done cautiously since different testing procedures were applied and pooling of data for age and gender was done differently across studies [12]–[14], [16], [17], [20]. Differences in testing procedures may contribute to the variability in distances walked in six minutes, such as influencing the motivation of the child with the use of a measuring wheel [14] or interfering with the test procedure by measuring additional variables [16]. The influence of track length on a child's 6MWD is also not clear from previous reports [1], [15]. Furthermore, the pooling of data from both genders [16], [20] may possibly explain the lower achieved distances in those studies, although reports on the influence of gender are conflicting [14]–[17], [20].

The sample sizes were highly variable as well, with some studies reporting data from very limited samples [15]. Finally, the different ethnical backgrounds as well as anthropometric differences may further explain some of the variability across the different studies [12], [15], [18].

This study was set up to collect data on 6MWT in typically developing children as a reference for DMD boys. In this progressive disease of childhood, muscle loss and functional decline occur against a background of growth and development, resulting in the observation of an increase in 6MWD in younger children with DMD despite progressive impairment [5]–[8]. Converting raw 6MWD values into percentage predicted values based on a reference equation derived from age- and height matched peers seems the most straightforward method to account for those maturational influences [9]. Henricson et al.[9] concluded that the equation of Geiger et al. [14] was the most appropriate for their American DMD boys because of the large age range, Caucasian ethnicity and absence of heart rate as an independent variable. This equation has been derived from data collected from 280 healthy Austrian boys, unequally distributed between age 3 and18 years, with the largest sample in the age category 12 to 15 years, using a slightly different testing procedure (use of a measuring wheel, running allowed in the younger ones, 20 m track). We questioned whether this equation would be the most accurate for our sample as well.

When implementing the data of our study into the Geiger equation [14], the mean predicted value was higher than the originally measured value. This overestimation was observed in all age categories. The same observation was made for the North African reference equation [12]. As the Geiger equation has yet been implemented in DMD studies, we refitted this equation to our population to obtain a more accurate prediction model based on a larger sample with a homogenous distribution across age categories and applying a methodology as currently used in clinical trials in DMD.

This study is prone to several possible limitations. First, the data were obtained by a team of three different testers. Nonetheless, to limit possible inter-rater variation, all evaluators were well trained and followed a standardized operational protocol. Secondly, this study did not investigate parameters of endurance such as heart rate at baseline and post exercise, a variable reported to correlate with 6MWD [17], [20]. However, this study was set up to explore variables that could be useful in relation to DMD. In this disease a higher baseline heart rate and stunted reactions of heart rate to exercise have been reported [3], impeding the use of those variables in equations derived from healthy boys. Finally, our data were limited to elementary school boys aged 5 to 12 years. Further studies are required to generate data in pubertal and adolescent boys, especially since loss of ambulation has shifted to an older age in the contemporary natural history of DMD boys under chronic steroid treatment [34].

Despite these limitations, this study was the first to establish normative data of the 6MWT in a large cohort (N = 442) of healthy boys (5–12 years) and this in relation to age and height, using the same methodology as currently used in DMD studies [3].The normal values were measured in narrow age subcategories of one year with each category containing minimum 48 children. This study provided age- and height specific centile curves which might be clinically useful to judge the performance of DMD boys, the evolution of the DMD disease and the response to therapeutic intervention. This study confirmed previous reports on correlations between 6MWD with age and anthropometric variables in young children. It was however the first study to explore relations between myometric variables and the 6MWT in a large cohort of healthy subjects. Our study indicated that those variables do influence the 6MWD, especially up to the age of 9, however further research is required to identify additional factors influencing the 6MWT, especially in the older age group. Finally, the Geiger prediction equation, which use has been advocated for DMD studies, was refitted to this large sample of boys aged 5 to 12 years.
